# Assessment of breast cancer opportunistic screening by clinical–pathological indicators: a population-based study

**DOI:** 10.1038/sj.bjc.6605378

**Published:** 2009-10-27

**Authors:** A Bordoni, N M Probst-Hensch, L Mazzucchelli, A Spitale

**Affiliations:** 1 Ticino Cancer Registry, Institute of Pathology, Via in Selva 24, Locarno CH-6600, Switzerland; 2 Department of Chronic Disease Epidemiology/NICER, ISPM Zurich, University of Zürich, Sumatrastrasse 30, Zurich CH-8006, Switzerland; 3 Institute of Pathology, Via in Selva 24, Locarno CH-6600, Switzerland

**Keywords:** breast cancer, opportunistic screening, cancer registry, DCIS, tumour size, histological grade

## Abstract

**Background:**

Although some clinical–pathological features of breast cancers, such as the incidence of ductal cancer *in situ* (DCIS) and the diameter of invasive tumours, are sensitive indicators of early detection, comprehensive population-based studies of opportunistic screening are needed.

**Methods:**

Cases of DCIS or invasive breast cancer diagnosed in 1996–2007 were identified from the Ticino Cancer Registry (south of Switzerland). Time trends of age-adjusted incidence and mortality, as well as main clinical–pathological features, such as tumour diameter, AJCC stage and histological grade, were analysed.

**Results:**

A total of 3047 incident cases of female breast cancer were identified. The proportion of DCIS with respect to invasive cases increased from 5.8% in the period 1996–2001 to 6.4% in the period 2002–2007. The median tumour size of invasive cancers decreased from 20 mm in 1996–2001 to 18 mm in 2002–2007 (*P*<0.0001). An increase in well/moderately differentiated invasive tumours, from 67% in the period 1996–2001 to 73% in 2002–2007 (*P*<0.001), was detected and resulted in an Annual Percentage Change of incidence of 2.8 (95% confidence interval: 1.3; 4.3).

**Conclusion:**

An opportunistic screening strategy can lead to an improvement of prognostic features at diagnosis, but these features are still less favourable than those achieved by organised screening programmes.

Owing to the difficulty in implementing primary preventive measures for breast cancer, secondary prevention is correspondingly important, aimed at maximising detection of small cancers. Early detection diminishes the likelihood of clinical symptoms, disease progression, recurrence, distant metastasis, and death from breast cancer (Tabar *et al*, 1999). Trials have concluded that mammography screening in women aged from 50 to 69 can reduce breast cancer mortality by 25–30% (Gotzsche and Nielsen, 2006). Although Swiss French-speaking regions are covered by organised screening, the German- and Italian-speaking regions have no such programme. In the Italian- and French-speaking parts, physician-prescribed mammography is more frequent than in the German-speaking parts (Keller *et al*, 2001). In the south of Switzerland, the proportion of women aged 50–64 years who had undergone at least one mammography examination increased from 85% in 2002 to 88% in 2007 (Federal Office of Statistics, 2008).

The current coexistence of systematic screening programmes and opportunistic screening strategies in Switzerland with its high-quality health-care system provides an opportunity to investigate the merits and drawbacks of the two approaches (Zwahlen *et al*, 2004). Although both methods seem effective in reducing breast cancer mortality, data on opportunistic screening are limited (de Gelder *et al*, 2009).

The aim of this ecological population-based study was to assess such indicators as incidence rates, percentage of small cancers, stage distribution at diagnosis, axillary lymph node status and corresponding time trends for newly detected breast cancers, and to compare our data with those from populations with programmed screening.

## Materials and methods

Patients with a primary diagnosis of ductal cancer *in situ* (DCIS) or invasive breast cancer diagnosed between 1996 and 2007 were selected from the Ticino Cancer Registry located in the south of Switzerland, where more than 80% of women aged 50–69 years regularly undergo mammography (Bordoni and Mazzola, 2007; Bordoni *et al*, 2009).

Topography and morphology classifications followed the International Classification of Diseases for Oncology (ICD-O-III) and the WHO Classification of Breast Tumours (Fritz *et al*, 2000; Tavassoli and Devilee, 2003). Case registration followed the International Agency for Research on Cancer (IARC) guidelines and European Network of Cancer Registries (ENCR) recommendations (Tyczynski *et al*, 2003).

*In situ* lobular neoplasia and non-epithelial cancers such as lymphoma, sarcoma and phyllodes tumours were excluded from the analysis.

Essential information was abstracted from pathology reports, including tumour diameter, lymph node status, stage group (AJCC, 5th and 6th editions), histological grade (Bloom-Richardson system and the Elston and Ellis modification), laterality and hormonal receptor status. DCIS were graded according to nuclear features (European Commission, 1996; Shoker and Sloane, 1999). Individuals who received preoperative/neoadjuvant treatment (less than 5%) were excluded from analyses of tumour stage and diameter. Breast cancers expressing ER and PR in less than 5% of neoplastic cells were considered as negative for hormone receptor expression (Spitale *et al*, 2009). All histopathological analyses were carried out by a single laboratory and evaluated on routinely collected tissues by the same group of pathologists to ensure reproducibility.

### Statistical analysis

Mean and median values were provided for quantitative variables, whereas proportions represented qualitative variables. Differences between two periods (1996–2001 *vs* 2002–2007) were evaluated using Student's T-test for continuous variables and *χ*^2^ or Fisher's exact test for discrete variables (Armitage *et al*, 2002). Age-standardised incidence and mortality rates were calculated by the direct method (Esteve *et al*, 1994). Incidence rates of invasive and *in situ* lesions were produced for different age groups, for the above two periods and for single calendar years. Trends in incidence and mortality were measured as the estimated annual percentage change (APC) and the 95% confidence intervals (CIs) (Kleinbaum *et al*, 1988). Joinpoints representing the years when the trend changed were identified (Kim *et al*, 2000; National Cancer Institute, April 2005). Time trends were also analysed for mean and median tumour diameter, AJCC stage distribution (considering the shift from advanced to early stages), as well as histological grade at diagnosis. Statistical significance was determined at *P*<0.05 and the SAS System Version 9.1 (SAS Institute Inc., Cary, NC) was used for analysis.

## Results

A total of 3047 incident cases of female breast cancer were identified in the study period, of which 187 (6. 1%) were DCIS and 2860 (93.9%) invasive carcinomas.

Patient and invasive tumour characteristics are summarised for the two periods in Table 1. The mean patient age was 63.0 years, with no significant change during the study. About 80% of cases were diagnosed after age 50 years, those with *in situ* cancer being significantly younger (60.9 years) than those with invasive cancer (*P*=0.0198, data not shown); the proportion of *in situ* cases was higher among patients aged 50–69 years (57.7%), with a lower proportion aged 70 years or over (24.1%).

Among invasive cancers, we found that the mean diameter (data available for 85% of cases) decreased by 9%, from 22.0±13.2 to 20.3±12.6 mm, between 1996–2001 and 2002–2007 (*P*=0.0008); the corresponding medians decreased by 11% from 20 to 18 mm (*P*<0.0001). A decreasing trend of mean (APC:−1.3; 95% CI: −2.1; −0.5) and median (APC:−1.5; 95% CI: −2.5; −0.4) tumour size was detected from 1996 to 2007. A similar but stronger pattern was observed in the age group 50–69 years, with a significant reduction in mean (APC: −2.1; 95% CI: −3.1; −1.1) and median size (APC: −2.5; 95% CI: −3.9; −1.1). We also noted an increase in the number of tumours with a diameter ⩽10 mm or 11–20 mm in the more recent period, whereas cases with a diameter greater than 20 mm declined, both at all ages (*P*=0.0441, Table 1) and in the age group 50–69 years (*P*=0.1821, data not shown).

Overall, 40% of invasive cancers were associated with lymph node metastases at diagnosis, the respective proportions being 43.4% in women younger than 50 years, 42.3% in the age group 50–69 years and 34.2% at age of 70 years or over (data not shown). Comparing the two periods, there was no significant change in the proportion with lymph node metastases compared with those with no nodal involvement, even after age stratification. The mean and median numbers of examined lymph nodes decreased significantly from 2001 onwards, with an APC equal to −10.5 (95% CI: −13.8; −7.0) and −18.7 (95% CI: −25.1; −11.8), respectively, the pattern being similar at the age of 50–69 years. Simultaneously, the use of sentinel node procedure increased from zero in 1996 to 60.4% in 2007 (data not shown). Only 6% of invasive cancers presented distant metastases at diagnosis with no significant trend over the study period.

**Figure 1:**
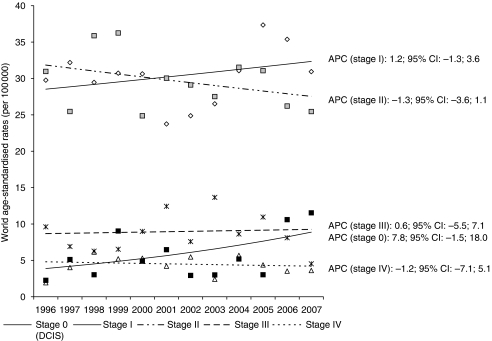
Trend of breast cancer incidence according to stage. Ticino (south of Switzerland), 1996–2007.

More than 80% of invasive cases were diagnosed at stage I or II. The proportion of women with stage I increased from 38.1% in 1996–2001 to 42.2% in 2002–2007 (Table 1) with similar results for ages 50–69 years, with a higher percentage of stage I cases in the last period (40.4% in 1996–2001 vs 45.7% in 2002–2007, *P*=0.3197). The results were confirmed when age-standardised incidence trends were observed (Figure 1).

**Figure 2:**
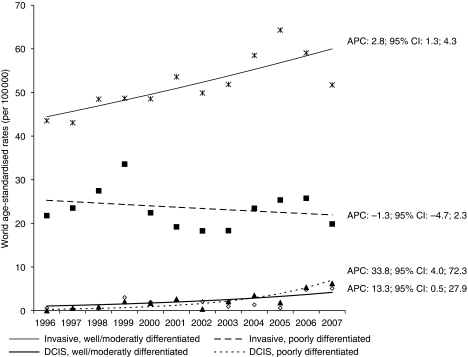
Incidence of invasive and *in situ* ductal breast cancers (DCIS), according to histological grade. Ticino (south of Switzerland), 1996–2007.

Well/moderately differentiated invasive lesions increased (from 67% in 1996–2001 to 73% in 2002–2007), whereas poorly differentiated cancers declined (*P*=0.0003); results for the age group 50–69 years were similar. An opposite but not significant trend was observed in the two periods for *in situ* lesions; a decrease of well/moderately differentiated cancers (from 58.3 to 44.3%) was accompanied by an increase in poorly differentiated cancers (from 41.7 to 55.7%). Considering the age-standardised incidence rates, we found a significant increase (APC: 2.8; 95% CI: 1.3; 4.3) in well/moderately differentiated invasive cancers and a decrease (APC: −1.3; 95% CI: −4.7; 2.3) in poorly differentiated invasive cancers (Figure 2). In DCIS cases, both well/moderately (APC: 13.3; 95% CI: 0.5; 27.9) and poorly (APC: 33.8; 95% CI: 4.0; 72.3) differentiated lesions increased.

Of the 2860 new cases of invasive breast cancer diagnosed in the period 1996–2007, 1273 (44.5%) occurred in women in the target age group (50–69 years) for mammography screening. The overall World age-standardised incidence rate was equal to 79.6 cases per 100 000 women with an APC of 0.9 (95% CI: −0.8; 2.7). The incidence trend in pre-menopausal women was substantially stable (APC: −0.7; 95% CI: −4.7; 3.5), whereas at ages 50–69 and 70+ years, an increasing trend was observed, though the estimated APCs were not statistically significant.

Of the 187 DCIS, 34 (18%) occurred before the age of 50 years, 108 (58%) at ages 50–69 years and the remaining 45 (24%) at ages 70+ years. The proportion of DCIS among the 3047 incident cases included in the study (DCIS and invasive tumours) increased from 5.8% in the period 1996–2001 to 6.4% in the period 2002–2007, and markedly in the final 2 years, when it reached 10%; at ages 50–69 years, this increased from 7.2% in 1996–2001 to 8.4% in 2002–2007 (data not shown).

Over the 12-year study period, the overall incidence of DCIS in Ticino was 5.7 cases per 100 000 women, and this remained essentially constant until 2005 (Figure 1). The final 2-year data (2006–2007), however, suggests an increase in world age-adjusted incidence (11.5 cases per 100 000), a pattern also observed after stratification by age group. The age-standardised incidence for DCIS among women in the target age group (50–69 years) increased between 1996–2001 and 2002–2007 (19.2 and 24.4 cases per 100 000, respectively); the rate for those aged 70 years or over also showed an upward trend (from 14.0 to 20.4 cases per 100 000).

The overall age-adjusted mortality rates of breast cancer in Ticino decreased from 20–22 cases in the 1980s to 14.6 cases per 100 000 in 2005, with a significant APC equal to −1.4 (95% CI: −2.4; −0.5). Mortality below age 50 years started to decrease significantly after 1998 with an APC equal to −19.2 (95% CI: −33.0; −2.6); a significant decrease was detected from 1990 in the age group 50–69 years (APC: −3.5; 95% CI: −6.0; −0.9); however, for women aged over 69 years, there was no change in breast cancer mortality over time.

## Discussion

The role of cancer registries in evaluating breast cancer screening has been highlighted in recent guidelines (Perry *et al*, 2008), and they can also provide population-based breast cancer data regardless of the mode of detection, whether by an organised screening programme or by opportunistic mammography or clinical means, thereby permitting an evaluation of a screening strategy for the whole population. Normally, essential quality parameters can be produced for an organised screening programme (Swedish Organised Service Screening Evaluation Group, 2007; NHS Breast Screening Programme, 2008; Perry *et al*, 2008), and cancer registries can also provide early indicators of screening efficacy, without waiting for the mortality data (Schaffer *et al*, 1996). We focused on specific indicators, and compared our results for an opportunistic screening with data obtained from other European and US population-based studies (Table 2). It is important here to stress that the different degrees of completeness, as well as age and ethnical distribution within these studies, have to be taken into account when interpreting differences between our and other findings.

Although not statistically significant, women overall, and particularly those aged 50–69 years, showed a downward stage shift, with an increase in stage I and a slight decrease in stage II cases compatible with what we expected from the literature (Coburn *et al*, 2004; Louwman *et al*, 2008). With respect to the change of TNM classification in 2003, no significant APC in the age-adjusted incidence trend according to stage was observed, as in other studies (Louwman *et al*, 2008), making it unlikely that the TNM change had influenced our results. The percentage of stage I cases (40.2% overall) was comparable to that in European regions with organised screening programmes (the Netherlands 39%, Denmark 43%), but was lower than that in Rhode Island, United States (53.5%) (Coburn *et al*, 2004; Jensen *et al*, 2008; Louwman *et al*, 2008).

The downward stage shift observed in southern Switzerland was accompanied by a decreasing trend of invasive tumour size at the time of diagnosis. The significant decrease in both median (*P*<0.0001) and mean (*P*=0.0008) diameter showed that early detection was also effective at this level. Taken together, the data suggest that an opportunistic screening here has been associated not only with an increased awareness of breast cancer in the population and among general practitioners, but also with the availability of high-quality diagnostic imaging. Nevertheless, the median and mean sizes are higher than those reported for Rhode Island, which has organised screening with 80% biannual attendance, (Table 2) (Coburn *et al*, 2004). Similarly, in Denmark, a median tumour with a diameter equal to 20 mm was observed in two regions where no organised screening was implemented, and that equal to 15 mm in a region where one was (Jensen *et al*, 2008). When we performed a subgroup analysis that considered tumour size for the period 2000–2005 (with the aim of making comparison with other studies without a period bias), the proportion of tumours with a diameter ⩽10 mm was 18.2% in Ticino, whereas in the Cantons of Geneva and Vaud, with population-based screening, this reached 26.1 and 30.1%, respectively (Table 2) (Schopper and de Wolf, 2007; Bulliard *et al*, 2009). In view of the difficulty in the pathological determination of the DCIS diameter, this was not analysed (Thomson *et al*, 2001; European Commission, 2006).

The trend, described above, of reduced tumour diameter at diagnosis should be biologically followed by a decrease in the detection of positive lymph nodes. However, we detected an essentially constant trend of positive lymph node incidence between 1996 and 2007. This finding could be attributed to competitive effects, such as the introduction of the sentinel lymph node procedure that has been adopted during recent years, as well as the improved histological workup associated with the multiple level investigation of lymph nodes, which has also been introduced by pathologists (Cserni, 1999; Kiaer *et al*, 2008; Madsen *et al*, 2008).

Although data are limited, poorly differentiated DCIS lesions (high grade) are probably associated with a significantly higher risk for invasive carcinoma (Vainio, 2002). High-grade DCIS shows abnormal mammography features more frequently than does low-grade DCIS, because of more obvious calcification. We observed an increase in poorly differentiated DCIS (Figure 2) that may reflect increased use of mammography and also could represent a downstaging from invasive to *in situ* cancers, as hypothesised (Sumner *et al*, 2007). We found an increasing trend of well/moderately differentiated invasive cases, accompanied by a decrease in high-grade cancers, that could be associated with early detection (Tabar *et al*, 1999; Schopper and de Wolf, 2007).

Ductal cancer *in situ* should increase when a screening programme is implemented (Miller *et al*, 2000; van Steenbergen *et al*, 2008) and increases have been reported from population-based studies in the United States, Australia, Italy and the Swiss Canton of Vaud (Levi *et al*, 1997; Barchielli *et al*, 1999; Kricker *et al*, 2004; Li *et al*, 2005). In southern Switzerland, we observed, among all tumours analysed (DCIS and invasive cases), a proportion of DCIS equal to 6.4% in the period 2002–2007, similar to that reported in two studies in the Netherlands (10% in 2000–2004 and 7.4% in 1984–2006), but our estimates are lower than in some US studies (13 and 15% in 1987–2001 and 1999–2005, respectively) (Table 2). Although a real difference in the incidence of breast lesions in Europe compared with North America cannot be excluded, it is important to highlight that the pathology criteria could be different. For the target age group 50–69 years, our observed 8.4% of *in situ* cancers is close, though not equal to what was reported from the Netherlands (11.6%), Geneva (12.3%) and Vaud (12.5%), where population screening programmes are implemented (Table 2). In the final 2 years of observation, the proportion of DCIS increased to 10%, along with the introduction of digital mammography and vacuum-assisted needle core biopsy (Thomson *et al*, 2001; Pisano *et al*, 2005).

Considering the mortality, both earlier diagnosis because of screening and better treatment may have had a role in the declining rate observed in the United States and some European countries (Schopper and de Wolf, 2009). A decreasing trend was reported in Switzerland generally, as well as in southern Switzerland for all ages and particularly in the age group 50–69 years, irrespective of the screening programme. Most likely, mortality as a quality indicator does not have enough resolution to discriminate between systematic and opportunistic screening.

Although the introduction of mass screening is the most likely explanation for the increased incidence of particularly early stage invasive breast cancer, increasing exposure to risk factors is also relevant (Louwman *et al*, 2008). Factors such as younger age at menarche, older age at menopause, older age at the birth of first child, lower parity, and shorter lactation have changed adversely over the past decade and so have probably contributed to the observed increase (MacMahon, 2006). Recently, the beginning of a decline in invasive cancers has been reported, attributed both to an increase in mammography-detected *in situ* cases and, particularly in the United States, a contemporaneous decline in the use of hormone replacement therapy (Jemal *et al*, 2007; Ravdin *et al*, 2007; Malmgren *et al*, 2008). However, the increasing trend of invasive breast cancers in the south of Switzerland, together with the stage shifting described above, along with tumour diameter decrease and *in situ* cancer increase, seems to be because of a progressive increase in the use of early detection diagnostics.

The increasing trend of positive prognostic factors shows that opportunistic mammography screening can make a substantial contribution to breast cancer diagnosis; yet the data also show that there is room for additional improvement in early detection in comparison with features achieved where organised screening is established.

## Conflict of interest

The authors declare no conflict of interest.

## Figures and Tables

**Table 1:** Clinical–pathological characteristics of patients with invasive breast cancers. Ticino (south of Switzerland), 1996–2001 *vs* 2002–2007

**Variable**	**All invasive cases, *n*=2860**	**1996–2001, *n*=1328 (46.4%)**	**2002–2007, *n*=1532 (53.6%)**	***P*-value**
*Age*				
Mean±s.d. (years)	63.0±14.5	62.7±14.8	63.3±14.3	0.2915
Median	63	62	63	0.1759
				
*Age-specific groups, n (%)*				
<50	591 (20.7)	284 (21.4)	307 (20.0)	0.6420
50–69	1273 (44.5)	582 (43.8)	691 (45.1)	
>69	996 (34.8)	462 (34.8)	534 (34.9)	
*Pre-menopausal (age⩽51), *n* (%)*	695 (24.3)	343 (25.8)	352 (22.9)	0.0762
*Post-menopausal (age>51), *n* (%)*	2164 (75.7)	985 (74.2)	1180 (77.0)	
				
*Basis of diagnosis*				
Non-microscopic	26 (0.9)	9 (0.7)	17 (1.1)	0.0021
Cytology	110 (3.8)	69 (5.2)	41 (2.7)	
Histology	2707 (94.7)	1240 (93.4)	1467 (95.8)	
DCO	17 (0.6)	10 (0.7)	7 (0.4)	
				
*Tumour size*				
Mean±s.d. (mm)	21.1±12.9	22.0±13.2	20.3±12.6	0.0008
Median	19	20	18	<0.0001
				
*Size-specific groups, n (%)*				
⩽1.0 cm	383 (15.8)	165 (14.6)	218 (16.9)	0.0441
1.0–2.0 cm	1082 (44.7)	486 (43.1)	596 (46.0)	
2.0–5.0 cm	888 (36.7)	446 (39.6)	442 (34.1)	
>5.0 cm	69 (2.8)	30 (2.7)	30 (3.0)	
Unknown or set after therapy	438	201	237	
				
*Lymph node status, n (%)*				
Positive	1032 (40.0)	491 (40.5)	541 (39.5)	0.5862
Negative	1551 (60.0)	721 (59.5)	830 (60.5)	
Missing or set after therapy	277			
				
*Clinical behaviour, n (%)*				
M0	2591 (94.0)	1197 (93.6)	1394 (94.3)	0.4230
M1	166 (6.0)	82 (6.4)	84 (5.7)	
Unknown	103	49	54	
				
*AJCC stage group, n (%)*				
Stage I	1023 (40.2)	464 (38.1)	559 (42.2)	0.2045
Stage II	1041 (40.9)	520 (42.7)	521 (39.3)	
Stage III	314 (12.4)	152 (12.5)	162 (12.2)	
Stage IV	166 (6.5)	82 (6.7)	84 (6.3)	
Unknown or set after therapy	316	110	206	
				
*Histological type, n (%)*				
Ductal	2308 (80.7)	1090 (82.1)	1218 (79.5)	0.0048
Lobular	309 (10.8)	121 (9.1)	188 (12.3)	
Mixed ductal and lobular	69 (2.4)	25 (1.9)	44 (2.9)	
Other	174 (6.1)	92 (6.9)	82 (5.3)	
				
*Histological grade (Elston/Ellis), n (%)*				
Well/moderately differentiated	1876 (70.4)	821 (67.0)	1055 (73.3)	0.0003
Poorly differentiated	789 (29.6)	405 (33.0)	384 (26.7)	
Unknown/unclassified	195	103	92	
				
*Laterality, n (%)*				
Right	1357 (48.3)	632 (48.2)	725 (48.3)	0.9624
Left	1453 (51.7)	678 (51.8)	775 (51.7)	
Unknown	50	18	32	
				
*ER status, n (%)*				
Positive	2095 (81.8)	874 (77.1)	1221 (85.4)	<0.0001
Negative	467 (18.2)	259 (22.9)	208 (14.6)	
Unknown	298	195	103	
				
*PR status, n (%)*				
Positive	1759 (68.8)	713 (63.2)	1046 (73.1)	<0.0001
Negative	799 (31.2)	415 (36.8)	384 (26.9)	
Unknown	302	200	102	

**Table 2:** Comparison of major indicators among screening programme guidelines, Ticino (south of Switzerland) data and other population-based studies

**Parameter**	**Screening Programme Guidelines**	**Ticino (south of Switzerland) 1996–2007**	**Other population-based studies** a
Proportion of *in situ* cancers	NA	6.1%	7.4 and 10% in the Netherlands^b^^,^^c^ 13 and 15% in the United States^d^^,^^e^
Proportion of *in situ* cancers (50–69 years)	10–20%	8.4%	11.6% in the Netherlands^c^ 12.3% in Geneva^f^^,^^g^ 12.5% in Vaud^f^^,^^g^
Proportion of invasive cancers with tumour size ⩽10 mm (50–69 years)	⩾25–30%	18.2%^h^	26.1% in Geneva^f^^,^^g^ 30.1% in Vaud^f^^,^^g^
Proportion of invasive cancers with tumour size ⩽20 mm (50–69 years)	NA	63.5%^h^	70.4% in Geneva^f^^,^^g^ 70.1% in Vaud^f^^,^^g^
Median tumour size for invasive cancers (mm)	NA	19 mm	15 mm in Rhode Island^d^ 15 mm in Denmark^i^ *20 mm in Denmark*^i^
Mean tumour size for invasive cancers (mm)	NA	21 mm	20 mm in Rhode Island^d^
Proportion of invasive cancers with negative lymph node	>70–75%	60%	53.7% in Denmark^i^ *43.3% in Denmark*^i^ 64.7% in Rhode Island^d^
Proportion of invasive tumours with stage I	NA	40.2%	39% in the Netherlands^b^ 43% in Denmark^i^ *29% in Denmark*^i^ 53.5% in Rhode Island^d^
Proportion of invasive tumours with stage II+	<25–30%	59.8%	57% in the Netherlands^b^ 46.5% in Rhode Island^d^

Abbreviation: NA=not available.

a All results come from regions where an organised screening programme is implemented, with the exception of those reported in italics, resulting from opportunistic screening.

b Louwman *et al* (2008).

c van Steenbergen *et al* (2008).

d Coburn *et al* (2004).

e Malmgren *et al* (2008).

f Bulliard *et al* (2009).

g Schopper and de Wolf (2007).

h Data for the period 2000–2005, with the aim of being comparable with other Swiss data (i.e., Geneva and Vaud).

i Jensen *et al* (2008).
